# Catatonia in autism and other neurodevelopmental disabilities: a state-of-the-art review

**DOI:** 10.1038/s44184-022-00012-9

**Published:** 2022-09-14

**Authors:** Shavon Moore, Debha N. Amatya, Michael M. Chu, Aaron D. Besterman

**Affiliations:** 1 University of California San Diego, Department of Psychiatry, San Diego, CA USA; 2 Rady Children’s Hospital San Diego, Division of Behavioral Health Services, San Diego, CA USA; 3 UCLA Semel Institute of Neuroscience and Human Behavior, Los Angeles, CA USA; 4 Children’s Hospital of Orange County, Division of Child and Adolescent Psychiatry, Orange, CA USA; 5 University of California Irvine, Department of Psychiatry, Irvine, CA USA; 6 Rady Children’s Institute for Genomic Medicine, San Diego, CA USA

**Keywords:** Human behaviour, Autism spectrum disorders, Clinical genetics, Immunopathogenesis

## Abstract

Individuals with neurodevelopmental disabilities (NDDs) may be at increased risk for catatonia, which can be an especially challenging condition to diagnose and treat. There may be symptom overlap between catatonia and NDD-associated behaviors, such as stereotypies. The diagnosis of catatonia should perhaps be adjusted to address symptom overlap and to include extreme behaviors observed in patients with NDDs, such as severe self-injury. Risk factors for catatonia in individuals with NDDs may include trauma and certain genetic variants, such as those that disrupt *SHANK3*. Common etiologic features between neurodevelopmental disabilities and catatonia, such as excitatory/inhibitory imbalance and neuroimmune dysfunction, may partially account for comorbidity. New approaches leveraging genetic testing and neuroimmunologic evaluation may allow for more precise diagnoses and effective treatments.

## Introduction

**Fig. 1:**
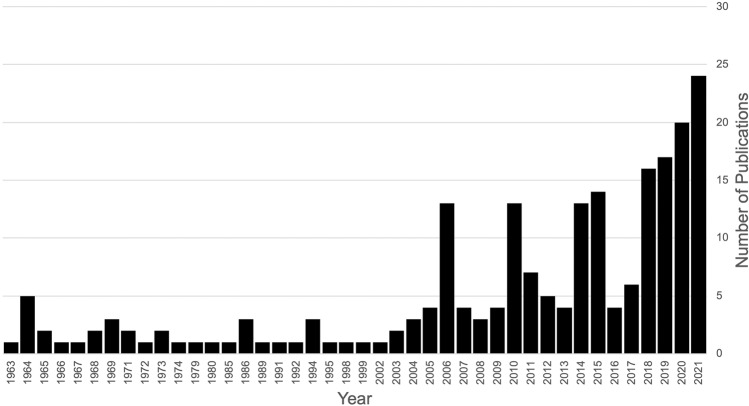
A recent increase in publications on catatonia in patients with NDDs. Data obtained from Timeline feature on https://pubmed.ncbi.nlm.nih.gov/ after searching “catatonia” and (“intellectual disability” or “developmental delay” or “autism” or “neurodevelopmental disorders”) on February 14, 2022.

Catatonia is defined as “…a marked decrease in reactivity to the environment” characterized by symptoms of negativism, mutism, stupor, excitement, repeated stereotyped movements, staring, and grimacing^1^. It can be a severely debilitating disorder and may occur at elevated rates in patients with neurodevelopmental disabilities (NDDs), such as autism spectrum disorders (ASD), developmental delay (DD), and intellectual disability (ID)^2^. Current prevalence estimates of catatonia in NDDs vary widely from 6 to 20.2%^3–6^. Likewise, prevalence estimates in neurotypical patients vary widely depending on study design (e.g., prospective vs retrospective), location of the study (e.g., country), recruitment site (e.g., medical or psychiatric hospital or community) and psychiatric or neurologic comorbidities. In a recent meta-analysis of neurotypical clinical samples, the mean prevalence of 74 studies was 9% with a range of 1.9–48.5%^7^. Given the wide range prevalence estimates for both populations, it remains uncertain if catatonia is more or less common in patients with NDDs than in neurotypical populations. However, anecdotally catatonia is being increasingly recognized by clinicians who work with patients with NDDs and publication trends on the topic reflect that (Fig. 1). This increased recognition led to catatonia being added as a modifier to ASD in Diagnostic and Statistical Manual 5^8^. So while it remains unconfirmed if individuals with NDDs have an elevated rates of catatonia, it is critical that we further our understanding of how neurobiological processes associated with NDDs may lead to a susceptibility to catatonia and what that might imply about how best to assess, diagnose, and treat catatonia in individuals with NDDs.

Many reports of patients with NDDs and catatonia discuss ASD or “autistic catatonia” specifically^9,10^. However, on close examination of these cases, many have complex neurodevelopmental presentations, often with low levels of premorbid functioning, suggestive of comorbid DD and/or ID in addition to ASD^11^ (Table 1). This entangling of NDD diagnoses makes it difficult to know if atypical neurodevelopment in the broadest sense, as compared to ASD-specific pathophysiology, may predispose individuals to catatonia.

**Table 1:** Summary of representative published cases of patients with NDDs and catatonia.

Reference	Age (years)	Symptoms	Trauma	Rating scale used	ASD	ID	Comorbidities	Genetic testing completed	ECT (sessions)	Maintenance ECT (months)
^106^	6	Mutism, appetite changes, withdrawal	+	−	+	−	ADHD	+		−
^107^	16	Withdrawal, mutism, regression, appetite changes, stupor, posturing	+	−	+	−	−	−	+	+ (6)
^107^	13	Stupor, grimacing, waxy flexibility, agitation,	+	−	+	+	−	+	+ (12)	−
^9^	15	Mutism, appetite changes, waxy flexibility, posturing, withdrawal	−	−	+	+	−	−	+ (-)	+ (-)
^9^	14	Stupor, posturing, waxy flexibility, echolalia, mutism, appetite changes, autonomic dysfunction	−	−	+	+	−	−	+ (12)	−
^9^	17	Self-injury, negativism, posturing, echolalia, echopraxia, Mutism	−	−	+	+	−	−	+	+ (-)
^108^	13	Catatonia, negativism, immobility, echolalia, posturing, and rigidity	−	−	+	−	−	−	−	−
^16^	11	Mutism, rigidity, posturing, waxy flexibility, appetite, regression	+	+	+	−	−	−	−	−
^109^	18	Self-injury, withdrawal, rigidity, staring, mutism, appetite changes, posturing	−	−	+	+	TS	+	+ (12)	+ (13)
^101^	8	Self-injury, stereotypy, agitation	−	−	+	+	−	−	+	−
^110^	14	Posturing, rigidity, waxy flexibility, regression, appetite changes, mutism, staring, autonomic instability	−	−	+	+	−	−	+ (12)	+
^111^	16	Agitation, echolalia self-injury, stupor, posturing	−	−	+	−	CVID, VWD	−	+ (29)	+
^111^	15	Agitation, self-injury, echolalia, stereotypy	−	−	+	+	MC, POS	−	+ (48)	+ (57)

+ = reported; − = not reported or unknown, *ASD* autism spectrum disorder, *ADHD* attention deficit hyperactivity disorder, *CVID* Common variable immune deficiency, *ECT* electroconvulsive therapy, *ID* intellectual disability, *MC* macrocephaly, *POS* polycystic ovary syndrome, *TS* Tourette syndrome, *VWD* Von Willebrand disease.

For the purposes of this review, we highlight key points using ASD as an example, with the same principles likely applying across NDDs. We describe what is known about catatonia in NDDs, discuss underlying neurobiological factors, and explore the complicated clinical and nosological overlap of catatonia with NDDs. Finally, we propose an assessment and management approach and suggest potential future strategies to improve the understanding and treatment of catatonia in patients with NDDs. While catatonia in ASD and NDDs has been reviewed numerous times before^12–17^, we hope that this review adds an integrative, biologically- and evidence-based, “state-of-the-art” perspective. We highlight what we believe are the greatest needs of future research on this topic and hope to set the field on an evidence-based path forward to improve assessment, diagnosis, and treatment of patients with catatonia and NDDs.

## Methods

We searched PubMed and Google Scholar using the terms: “catatonia” and (“intellectual disability” or “developmental delay” or “autism” or “neurodevelopmental disorders”) on February 14, 2022. We subsequently checked reference lists of articles and reviews until we were no longer identifying new sources. Literature published in English was selected for relevance to the topics of this Review (e.g., history, epidemiology, clinical features, treatment).

## Results

### History

In the study of developmental psychopathology, there has long been a debate about the proper classification of certain overlapping classes of symptoms—catatonic, autistic, and psychotic—dubbed “the iron triangle”^18^. Early physicians had different approaches to classifying patients with a mix of psychomotor symptoms, deficits in social and language skills, and psychotic symptoms. For example, in the early 20th century the term “childhood schizophrenia” was widely used to describe a wide range of patients with a mix of these symptoms, to the point that it became so non-specific that Sir Michael Rutter urged the term to be fully retired to “the history of psychiatry” in 1972^19^. He similarly argued for the separation of a disorder fully distinct from psychosis or schizophrenia termed autism or “infantile autism”, characterized by “failure of social development (of a specific type), a deviant and delayed language development, and various ritualistic activities”^19^. Karl Leonhard took a different approach to categorizing symptoms of “…stereotypies, impulsive-aggressive, self-injurious and disruptive behaviors, lack of expression, negativism, excitement, ambivalence, counter-grasping, mannerisms, peculiar speech patterns including echolalia, and neologisms…”. He classified these symptoms as “early childhood catatonia”, a subtype of “psychomotor psychoses”^20^. This alternative formulation through the lense of catatonia, as opposed to autism, is reflective of the overlapping symptamotolgy between these disorders. Nearly a century later, there are clearer distinction between what constitutes psychosis, autism, and catatonia, but there are certainly cases where it remains challenging to understand if new psychomotor or negativistic symptoms represent an exacerbation of preexisting neuropsychiatric symptoms or comorbid catatonia in patients with NDDs^6^. Thankfully, there is increasing recognition of the comorbidity between catatonia and NDDs^21^ and a growing body of literature on overlap of these conditions (Fig. 1).

### Phenomenology

“Classic” catatonia can be observed in patients with NDDs and may at times be straightforward to recognize and treat. However, there is often significant overlap in symptomatology between catatonic symptoms and some behaviors commonly seen in patients with NDDs. These include motor stereotypies, mannerisms, rituals, mutism, echolalia, and negativism^10^, which can make it challenging to distinguish an exacerbation of previous behaviors from catatonia. It is also unclear if the definition of catatonia in patients with NDDs should be altered to incorporate their neurodiversity. For example, it has been suggested that “autistic catatonia” may be best defined by freezing when carrying out actions, resistance to prompting, slow voluntary motor movements, and stopping in the course of movement^22^. However, this definition may capture behaviors nonspecific to catatonia and lead to overdiagnosis. It has also been suggested that extreme behaviors such as severe self-injury or unremitting tics may be uniquely indicative of catatonia in individuals with NDDs^14,23^. These behaviors can result in severe morbidity and impairment, and can be refractory to standard treatments, and are sometimes responsive to catatonia treatments like benzodiazepines and electroconvulsive therapy (ECT)^14^ However, it remains to be demonstrated whether these behaviors represent their own clinical entity or are part of the catatonia continuum for patients with NDDs. Currently, only clinical reports (and not rigorous field trials) support these behaviors as part of the catatonia syndrome in patients with NDDs, which creates the risk of confirmation and reporting bias^24,25^. Therefore, a rigorous assessment of the validity and reliability of expanded criteria for catatonia in NDDs is warranted.

### Trauma

Trauma has long been recognized as a potential risk factor for catatonia or catatonia-like states^26^. For example, freeze responses are observed in individuals with post-traumatic stress disorders, victims of violent assault, and those who have experienced urban violence, environmental disasters, and military combat and may represent a primitive human response to imminent doom and danger^27^. Likewise, a catatonic-like response to trauma and stress, defined by significant reduction in oral intake, communication, movement, and self-care, termed “Pervasive Refusal Disorder” has been described in neurotypical patients of asylum-seeking families^28^. It remains controversial whether this disorder is nosologically separate from catatonia^29^.

**Fig. 2:**
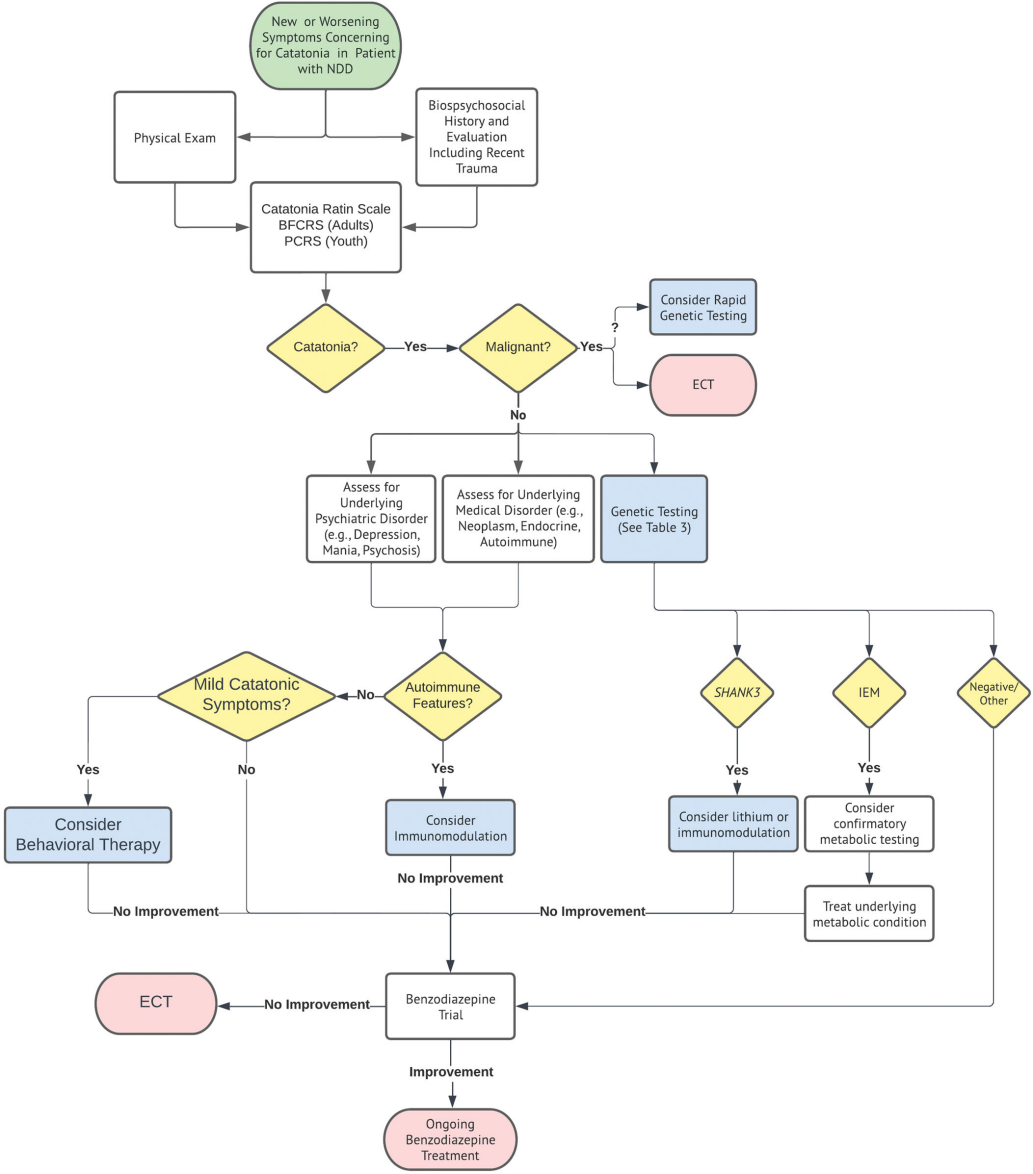
Proposed evaluation and treatment of catatonia in patients with NDDs. BFCRS Bush-Francis catatonia rating scale, ECT electroconvulsive therapy, IEM inborn error of metabolism, PCRS Pediatric catatonia rating scale, Green circles represent entry points, Red circles represent exit points, Yellow diamonds represent decision points, White boxes represent established processes, Blue boxes represent processes novel to our algorithm, “?” represents a potentially informative approach to consider but without sufficient data to fully support implementation.

In patients with NDDs who may be exquisitely sensitive to their environment, disruptions to important daily routines or losses of important life figures can be highly distressing, even traumatic, and potentially triggering of catatonia. In an early study (using a very broad definition of catatonia), 2.5% of patients with NDDs and catatonia had precipitating stressors such as grief, pressures at school, and loss of structure after the environmental change (Wing & Shah, 2000). The true rate of trauma precipitating catatonia is likely higher, based on recent clinical experience and published reports (Table 1). Other traumatic experiences, such as physical abuse and parental divorce, have been associated with the emergence of catatonic symptoms in patients with ASD^12,26^. Although there are no current guidelines regarding the evaluation of trauma in catatonia, it may be worth considering screening patients with NDDs presenting with catatonic symptoms for recent stressors or traumas to aid diagnosis and potentially guide treatment (Fig. 2). Addressing the trauma through behavioral or other psychotherapeutic approaches may ameliorate catatonic symptoms, but that is yet to be rigorously assessed.

### Assessment

The heterogeneous nature of catatonia and the overlapping symptomatology with NDDs makes it challenging to create a universally generalizable and valid standardized assessment tool for all patient populations. As a results, several catatonia rating scales have been shown to have fairly low specificity^30^. The Bush-Francis Catatonia Rating Scale (BFCRS) is the most widely used validated catatonia rating scale in neurotypical adults and has been used in patients with NDDs as well^31^. An adaptation of the BFCRS, the Pediatric Catatonia Rating Scale (PCRS), is validated for pediatric patients in the inpatient setting that captures most of the same features as the BFCRS, with the exception of hyperactivity/excitement^32^. The Attenuated Behavior Questionnaire (ABQ)^33^ was developed to specifically capture the “autistic catatonia” syndrome in the outpatient setting that was previously described^3^. It is a 34-item, questionnaire that can be administered to caregivers of patients with ASD that has some discriminant validity for catatonia in this population^33^. However, the ABQ may over-estimate the prevalence of catatonia in this population (up to 48% in the original study) and likely has low specificity, given ABQ scores are positively correlated with measures of depression and repetitive/restrictive behaviors^33^. Therefore, the BFCRS and PCRS remain the most reliable scales to assess for catatonia in adult and pediatric patients with NDDs, respectively, and their regular implementation may help improve the consistency of future studies to improve comparability (Fig. 2).

### Pathophysiology

The etiology of catatonia remains poorly understood^34^. However, the potentially elevated rates of catatonia in patients with NDDs and especially the overlapping symptomatology of catatonia and ASD, such as repetitive behaviors, stereotypic speech, and mannerisms, suggest that there may be common biological mechanisms underlying these conditions. Biological convergence could occur at multiple levels, including the genetic level, the neural circuit level, and/or the neuroimmune interface level (Table 2). Improving our understanding of the neurobiological overlap between NDDs and catatonia on these different scales may help us make sense of the diversity of catatonia presentations in patients with NDDs.

**Table 2:** Examples of potential convergent biological mechanisms in catatonia and NDDs.

Level	Mechanism	Catatonia	NDDs
Genetic	Noncoding single nucleotide variants	• Small nucleolar RNA mutations are hypothesized to impact downstream gene regulation & splicing in catatonia^45^	• Mouse models with small nucleolar RNA deletions recapitulate symptoms of autism^46^
	Structural variants	• 22q13.3 and 22q11.2 deletions are associated with catatonia^39^	• Disruption of 22q13.3 and 22q11.2 deletions associated with NDDs^112^
Neural circuits	EI imbalance	• Local glutamate overactivity and GABA underactivity is thought to play a role in catatonia^34,52^	• Mouse models support disruption in EI balance leading to ASD phenotypes. Increasing EI balance in prefrontal cortex using optogenetics leads to social deficits^61,62^ • Decreased GABA receptor density and altered GAD1 and GAD2 levels. Functional imaging studies identify local hyperconnectivity and decreased long-range connections^60^
Neuroimmune interface	Autoimmunity	• NMDAR encephalitis is causally linked to the development of catatonia^52^ • Systemic autoimmune conditions, like SLE, increase risk for the development of catatonia^113^ • Rodent models suggest that brain inflammation in catatonia is mediated by microglial activation^37^ • Standard treatments for catatonia, like ECT, have been shown to have an immunomodulatory effect over chronic administration^71^	• Family history of autoimmune conditions increase the risk for ASD^65^ • Gene network analysis identify immune dysregulation and microglial activation as key molecular signatures^77^ • Rodent models suggest that attenuation of microglial activity can rescue ASD-like behaviors^37^

*ASD* autism spectrum disorder, *EI* excitatory/inhibitory, *NMDAR* NMDA receptor, *NDDs* neurodevelopmental disabilities, *SLE* systemic lupus erythematosus.

The degree of familial aggregation^35^ of catatonia (e.g., the degree to which catatonia phenotypes tend to cluster in families that cannot be accounted for by chance) varies significantly depending on how it is defined, but overall points to a strong genetic contribution^36^. Candidate genes (e.g., genes hypothesized to be associated with a disorder based on their biological function) such as 2′,*3*′-*cyclic nucleotide 3*′-*phosphodiesterase* and *myelin basic protein* have been implicated in catatonia through animal studies of catatonia-like behaviors and correlation of candidate genotypes with catatonia symptoms in patients with schizophrenia^37^. However, there are significant limitations to candidate gene studies in psychiatry^38^, and these associations have not, to our knowledge, been replicated in large-scale population-based genetic studies. Catatonia has been observed in patients with NDDs with established genetic etiologies^39^, such as Prader-Willi syndrome^40^, 22q13.3 deletion syndrome (Phelan-McDermid syndrome)^41^, Down syndrome^42^, 22q11.2 Deletion syndrome^43^, and late-onset Tay-Sachs disease^44^. These rare genetic disorders can shed light on the neurobiological etiology of catatonia. For example, it has been proposed that many of the genetic disorders above have convergent neurobiological disruption of the brain-specific, non-coding micro-RNA, *SNORD115*, that lies in the Prader-Willi/Angelman syndrome region (e.g., 15q11. 2 - q13) and plays a central role in nucleolar function^45,46^. SNORD115 is known to regulate the downstream effector genes of Ral GEF With PH Domain And SH3 Binding Motif 1 (*RALPGS1*), which subsequently modulates RAS Like Proto-Oncogenes A and B (RalA and RalB) and their binding proteins^45,47^. This may lead to NMDA receptor dysfunction in the postsynaptic density through altered endocytotic and autophagic lysosomal degradation^45^. NMDA receptor dysfunction is likewise hypothesized to underlie catatonia risk associated with *SHANK3*-associated disorders as it leads to alterations in cellular excitability^45,48^. There is potential mechanistic overlap with an ASD susceptibility locus linked with catatonia, 15q15-q21, which includes genes responsible for encoding GABA receptors B3, A5, and G3^49,50^, again suggesting dysregulated cellular excitability may also be contributory to the pathogenesis of catatonia. This genetic evidence suggests that both catatonia and ASD may be associated with changes in Excitatory-Inhibitory (EI) balance, a circuit level dysfunction that has been observed both in animal models of catatonia and clinical settings^51^.

EI balance has been defined as, “…a stable global level of activity within a particular circuit, even though individual groups of neurons may exhibit transient imbalances, and these groups of neurons can be dynamic over time”^51^. A pathological EI imbalance that may be present in catatonia can be mediated through increased excitatory tone by local states of low dopamine, which can trigger excess glutamate release and excitotoxicity^34,52^. The interaction between dopamine and catatonia is further strengthened by the observation that dopamine blockade with antipsychotics can exacerbate symptoms of catatonia, and NMDA receptor blockade can conversely relieve symptoms^11^. Similarly, decreased inhibitory tone through GABA hypoactivity may play an important mechanistic role in catatonia^53,54^ that can be ameliorated through the use of GABA-ergic medications like benzodiazepines and through ECT^12,26^. Neuroanatomical correlates of EI dysregulation in catatonia have been studied with neuroimaging. Hypokinetic catatonia has been associated with localized hyperactivity in the supplementary and pre-supplementary motor areas, which are important in the generation and regulation of movement through interaction with the basal ganglia^11,34^. GABA concentration specifically within the supplementary motor areas are correlated to individual differences in motor planning, thus emphasizing the role for local EI imbalance within these particular brain structures as a potential important mechanism in catatonia and source of heterogeneity in clinical manifestation^55^.

Similarly, EI imbalance has been implicated in the pathogenesis of ASD^51^. From the integration of post-mortem and genetic analyses, it has been suggested that inhibitory hypofunction is a potential convergent pathway mechanism for the diverse pool of genetic mutations associated with synaptic dysfunction in ASD^56,57^. This may manifest as reduced synaptic input into inhibitory neurons, as is the case for variation in ASD-associated genes, *NLGN3* and *MECP2*, or diminished inhibitory output on excitatory circuits, which may occur secondary to *SHANK3* or *TSC1* dysfunction^58,59^. Furthermore, reduced GABA receptor density has been observed in individuals with ASD, consistent with a theory of reduced inhibitory tone and subsequent EI imbalance^60^. This finding has been recapitulated in rodent models, where optogenetic modulation of cortical EI precipitates ASD-like social deficits, which are rescued by GABA agonism or NMDA antagonism^61,62^. On a clinical and epidemiological level, EI imbalance across brain regions is consistent with the high comorbidity between ASD and epilepsy, a disorder characterized by extreme excess of neural excitation or deficit of inhibition^63^. Therefore, the strong evidence for loss of inhibitory neural circuit tone in both ASD and catatonia may represent a convergent mechanism between the two disorders.

The neuroimmune axis refers to the flow of information between the immune and nervous systems that play a role in both homeostasis and a variety of disease states^64^. Neuroimmune dysfunction, particularly through autoimmune activation, has also been associated with both ASD and catatonia^65,66^. For example, autoimmune encephalitis (AIEs) of various etiologies (e.g., Anti-NMDA receptor (NMDAR); Anti-GABA-A receptor) have been observed in patients with catatonia^67,68^. Inflammatory physiologic disorders (e.g., systemic lupus erythematosus and thyroid disorders) have also been described in patients with catatonia^66,69^. NMDAR encephalitis is the autoimmune disorder most frequently associated with catatonia and serves as a potential link between neuroimmune interactions and EI balance, as discussed above^66^. In NMDAR encephalitis, autoantibodies target subunits on the receptors of excitatory glutamatergic neurons, causing internalization of the receptor, excitatory hypofunction, and a host of neuropsychiatric symptoms, such as psychosis, seizures, impaired cognition, and catatonia^69^. Interestingly, catatonia associated with NMDAR encephalitis has been effectively treated with both benzodiazepines and ECT, suggesting that strong neuromodulation may regulate immune dysfunction^66^. However, the impact of ECT on the neuroimmune system is complex, with some conflicting outcomes and variability depending on the chronicity of treatment and the model used. For example, a transient upregulation of neuroinflammatory cytokines, microglial activation, and an increase in the oxidative stress response are observed following acute ECT in animal models^70^. But in a rodent model of autoimmune encephalomyelitis, electroconvulsive seizures attenuate the innate immune system through reduced activation of T cells by microglia over time^71^. Therefore, repeated ECT treatments may directly counter the pro-inflammatory effects of AIE through modulation of the innate immune system in brain specific immune cell types^71^. These findings highlight the complex relationship between neuroimmunity, neuroinflammation, and the incompletely understood mechanisms of current catatonia treatments.

Similarly, the development of ASD has been linked to both acquired and inherited immune dysregulation^72^. Patients with ASD are more likely to have a family history of autoimmune conditions, such as type 1 diabetes, psoriasis, hypothyroidism, and rheumatoid arthritis^73^. A subset of mothers may pass maternal antibodies to their fetus during prenatal development, which has been linked to macrocephaly and ASD-related traits, such as reduced language expressivity and stereotypic behaviors^73,74^. Additionally, a study of monozygotic twins discordant for ASD revealed that the top differentially expressed gene across pairs was *IGHG4*, which codes for a surface protein on B-cells that plays a key role in immune cell activation^75^. This adds to a growing body of literature that demonstrates the importance of immune and inflammatory genes in ASD, such as reduced expression of genes in the complement pathway^76^, identification of gene networks of microglial activation that may impact synaptic transmission^77^, and differential impacts of microglial protein synthesis on synaptic pruning and ASD related behaviors across sexes^78^. Later in life, individuals with ASD are prone to developing comorbid autoimmune conditions at elevated rates, particularly with conditions that affect the nervous system, such as multiple sclerosis, myasthenia gravis, and Guillain-Barré syndrome^79^. Therefore, immune dysregulation through inherited and acquired factors likely plays a significant role in the pathophysiology of ASD.

### Diagnosis

Genetic testing and counseling is now recommended in the evaluation of all patients with NDDs (with or without catatonia), because of the high diagnostic yield (~35%) and increasing promise of diagnostic clarity, medical prognosis, and value to the patient and family^80^ (Table 3). For catatonia in patients with NDDs, genetic testing can provide additional information about the underlying etiology and even suggest specific treatments if an inborn error of metabolism, like late-onset Tay-Sachs disease^44^ or a *SHANK3*-related disorder are detected^81,82^. However, standard genetic testing and counseling approaches typically take weeks or months due to limited genetics providers, insurance authorization and the testing process itself. For critically ill patients with debilitating catatonia, this timeline may be insufficient. Rapid genetic testing, notably rapid whole genome sequencing (rWGS), with turnaround times of hours to days, has been shown to improve outcomes and reduce medical expenses for pediatric and neonatal intensive care patients^83^. It may be worth considering for the most emergent cases of catatonia, especially malignant catatonia or severe self-injury (Fig. 2). For less critical patients, standard genetic testing approaches should include methods that at least detect large genomic deletions and duplications (e.g., chromosomal microarray) and single base pair changes (e.g., exome sequencing) or those that detect both variants together (e.g., WGS), as both variant types have been associated with NDDs and catatonia^39,84^ (Table 3 and Fig. 2). Appropriate genetic counseling before ordering the tests and returning the results is essential to ensure informed consent/assent and optimal outcomes^84,85^.

**Table 3:** Genetic testing strategies for neurodevelopmental disabilities.

**Fragile X testing**
Previously recommended as first-tier test for all males with NDDs, but low yield has resulted in reconsideration of this recommendation (https://www.nature.com/articles/gim2017146)
Uses polymerase chain reaction to detect CGG repeats in the *FMR1* gene
>200 repeats cause Fragile X Syndrome, while 55–200 repeats is associated with Fragile X premutation-associated conditions (https://www.futuremedicine.com/doi/full/10.2217/fnl.14.11)
**Chromosomal microarray**
Detects genomic deletions and duplications as seen in 22q11.2 deletion syndrome or 15q11.2 duplication
Considered a first-tier test for NDDs with diagnostic yield of 10–20% (https://www.sciencedirect.com/science/article/pii/S0002929710002089)
**Gene panel testing**
Varies widely depending on the specific company
Detects a limited range of genetic variants known to be associated with NDDs
**Whole exome sequencing**
Uses next-generation sequencing to identify single base pair changes (single nucleotide variants) in gene-coding regions (exons)
Recent recommendations to include as first- or second-tier test for NDDs with diagnostic yields up to ~50% in most severely affected patients^80^.
Does not detect genomic deletions/duplications or repeat expansions
**Whole genome sequencing**
Uses next-generation sequencing to identify single base pair changes (single nucleotide variants) in both gene-coding regions (exons) and gene regulatory regions (introns)
Can detect genomic deletions/duplications, but not repeat expansions
Recent recommendations to include as first- or second-tier test for NDDs with diagnostic yields up to ~50% in most severely affected patients^80^.
**DNA methylation analysis**
For a few NDDs such as Beckwith-Wiedemann, Prader-Willi, and Angelman syndromes, abnormal methylation patterns are causative (https://pubmed.ncbi.nlm.nih.gov/28818477/)
Usually only ordered when clinically suspected

AIEs such as NMDAR encephalitis, should be considered in any patient with NDDs presenting with catatonia. A standard clinical assessment looking for key features of AIEs including autonomic dysfunction, new-onset seizures, and new focal neurologic disorders, should always be performed^69^. However, these symptoms can sometimes be challenging to differentiate from exacerbations of pre-existing behaviors or neurologic conditions in patients with NDDs. For example, repetitive behaviors can intensify in severity that may be interpreted as hyperactive catatonia and some epilepsy syndromes can present in adolescence^86^, which could be mistaken for a symptom of AIE. When the rate of onset of these symptoms is rapid or subacute, an AIE should be strongly considered and a neuroimmunologic workup, including serum and cerebral spinal fluid studies for anti-neuronal autoantibodies, should be initiated^87–89^. If there is strong suspicion for an AIE in a patient with catatonia, immunomodulatory treatments, like intravenous immunoglobulins or systemic glucocorticoids^87–89^ may be considered prior to benzodiazepine or ECT treatment in order to target the underlying cause (Fig. 2). Caution should be taken in basing the diagnosis of catatonia off of treatment response alone, as individuals with catatonia due to an underlying AIE may respond to benzodiazepines or ECT treatment, but this may preclude the initiation of definitive immunomodulatory management and could result in poor long-term outcomes^66^. Furthermore, the diagnosis of AIE should trigger an evaluation for malignancy, as these conditions can occur as part of a paraneoplastic syndrome^90^.

Immunomodulatory therapies are also being actively explored in the treatment of ASD. Minocycline and Vitamin D have been shown to modulate microglial activation and improve ASD-related behaviors in animal models^91,92^. Given the possibility of some convergent neuroimmune mechanisms involving microglia, the question arises of whether patients with co-occurring NDD and catatonia may benefit more than neurotypical peers from immunomodulatory treatment. Four patients were recently described with *SHANK3*-associated NDDs (including but not limited to ASD) and comorbid neuropsychiatric symptoms, including catatonia^82^. They were treatment-resistant to standard psychotropics, but were rapidly responsive to immunomodulatory treatments, such as intravenous immunoglobulins, mycophenolate, and rituximab^82^. Larger, randomized trials are needed to clarify the role of these medications in treating patients with NDDs and catatonia, but there is growing evidence that they may especially benefit from expedient neuroimmunologic evaluation and treatment.

### Treatment

There is a dearth of research on the behavioral interventions for catatonia in patients with NDDs. Behavioral interventions targeting sensory, perceptual, and neurocognitive functioning have been attempted with mixed results^10^, but with some recent success using a “prompt-fading” approach, where behavioral interventions are slowly reduced overtime until the patient is able to function more independently^93,94^. Reducing stressors, providing structure throughout the day with activities that bring the patient joy, and using prompts to overcome movement difficulties improved clinician-reported ratings of movement, response, and functional independence^3,95^. These studies suggest that behavioral therapies may have a role in improving symptoms of mild catatonia in patients with NDDs or augmenting other treatments, but there is currently insufficient data to recommend behavioral interventions as a primary treatment approach.

The use of medications for the treatment of catatonia in neurotypical patients and patients with NDDs and catatonia follows a similar path. The gold standard pharmacotherapy continues to be benzodiazepines, such as lorazepam in doses ranging from 6–24 mg^96,97^. Second- and third-line agents, such as zolpidem or amantadine, for neurotypical adults currently have no safety or efficacy data in patients with NDDs and catatonia. There are preliminary reports of genetically guided treatment for catatonia, where patients with *SHANK3* variants and catatonia responding positively to lithium therapy^81,98^ and to immunomodulation^82^.

For patients with catatonia that see little or no benefit from treatment with benzodiazepines, ECT remains the gold standard^99^. An especially high ethical bar must be met for providing ECT to patients with NDDs and catatonia, as they often cannot provide informed consent either due to their baseline intellectual disability, their acute psychiatric state, or both, and are thus highly vulnerable^100^. ECT might be considered earlier in the treatment algorithm if the situation is emergent, such as in the case of malignant catatonia (Fig. 2). While it is not definitively established if severe SIBs are nosologically separate from catatonia, ECT can be an effective treatment for both conditions. For example, a patient with an NDD who had 100 self-injurious episodes per hour was diagnosed with hyperactive catatonia and treated with ECT 3×/week for 5 weeks, resulting in a decreased number of episodes to 20 per hour^101^.

## Discussion

We share our perspective on some of the most important recent advances in the understanding of catatonia in patients with NDDs and highlight some of the biggest gaps in our knowledge in this state-of-the-art (non-systematic) review. While we made every effort to be comprehensive, inclusive, and unbiased in our literature search and analysis, some work invariably was given more weight than others, which is a limitation. We synthesize the current literature and propose a novel evaluation and treatment algorithm (Fig. 2) that has some similarities to previous recommendations^12^, but with important advancements (highlighted in blue) leveraging the most recent developments in our understanding of the behavioral, genetic, and autoimmune bases of NDDs and catatonia. We envision a future in which patients with NDDs impacted by catatonia may undergo a rapid molecular evaluation that has the potential to yield both diagnostic information and inform precision treatment plans. Due to the significant morbidity associated with severe forms of catatonia, patients stand to benefit from the rapidly improving turnaround time for genomic and serological analyses^102^.

A significant future challenge will be figuring out how to further refine and validate the diagnosis of catatonia for patients with NDDs, given the heterogenous presentations along with symptom overlap. The use of standardized scales in assessment and case reporting could help improve comparability of cases and aid our understanding of the progression of catatonia in the NDD population. Rigorous, prospective, multi-institutional studies will be necessary to recruit sufficiently large cohorts of patients to address these questions. As has been demonstrated for NDDs in general, a robust understanding of genetic risk factors for catatonia in NDDs could shed light on the neurobiological underpinnings^103^. Epigenetic studies may shed light on how psychological distress and trauma can shift neurocircuitry towards a state of catatonia in individuals with NDDs^104^. The use of animal or organoid models of genetic syndromes known to predispose to catatonia, such as 22q13.3 deletion syndrome or Prader–Willi Syndromes, may be particularly illuminating, as they may provide insights that generalize to idiopathic cases of NDDs^105^.

## Data Availability

All data from this publication is freely available upon request

## Code availability

There is no code associated with this publication.
